# Scholarly Influence of the Conference and Labs of the Evaluation Forum eHealth Initiative: Review and Bibliometric Study of the 2012 to 2017 Outcomes

**DOI:** 10.2196/10961

**Published:** 2018-07-27

**Authors:** Hanna Suominen, Liadh Kelly, Lorraine Goeuriot

**Affiliations:** ^1^ Research School of Computer Science College of Engineering and Computer Science The Australian National University Canberra, ACT Australia; ^2^ Machine Learning Research Group Data61 Commonwealth Scientific and Industrial Research Organisation Canberra, ACT Australia; ^3^ Faculty of Science and Technology University of Canberra Canberra, ACT Australia; ^4^ Department of Future Technologies Faculty of Science and Engineering University of Turku Turku Finland; ^5^ Department of Computer Science Maynooth University Maynooth, Co Kildare Ireland; ^6^ Grenoble Informatics Laboratory Université Grenoble Alpes Grenoble France

**Keywords:** evaluation studies as topic, health records, information extraction, information storage and retrieval, information visualization, patient education as topic, speech recognition, systematic reviews, test-set generation, text classification

## Abstract

**Background:**

The eHealth initiative of the Conference and Labs of the Evaluation Forum (CLEF) has aimed since 2012 to provide researchers working on health text analytics with annual workshops, shared development challenges and tasks, benchmark datasets, and software for processing and evaluation. In 2012, it ran as a scientific workshop with the aim of establishing an evaluation lab, and since 2013, this annual workshop has been supplemented with 3 or more preceding labs each year. An evaluation lab is an activity where the participating individuals or teams’ goal is to solve the same problem, typically using the same dataset in a given time frame. The overall purpose of this initiative is to support patients, their next of kin, clinical staff, health scientists, and health care policy makers in accessing, understanding, using, and authoring health information in a multilingual setting. In the CLEF eHealth 2013 to 2017 installations, the aim was to address patient-centric text processing. From 2015, the scope was also extended to aid both patients’ understanding and clinicians’ authoring of various types of medical content. CLEF eHealth 2017 introduced a new pilot task on technology-assisted reviews (TARs) in empirical medicine in order to support health scientists and health care policymakers’ information access.

**Objectives:**

This original research paper reports on the outcomes of the first 6 installations of CLEF eHealth from 2012 to 2017. The focus is on measuring and analyzing the scholarly influence by reviewing CLEF eHealth papers and their citations.

**Methods:**

A review and bibliometric study of the CLEF eHealth proceedings, working notes, and author-declared paper extensions were conducted. Citation content analysis was used for the publications and their citations collected from Google Scholar.

**Results:**

As many as 718 teams registered their interest in the tasks, leading to 130 teams submitting to the 15 tasks. A total of 184 papers using CLEF eHealth data generated 1299 citations, yielding a total scholarly citation influence of almost 963,000 citations for the 741 coauthors, and included authors from 33 countries across the world. Eight tasks produced statistically significant improvements (2, 3, and 3 times with *P*<.001, *P*=.009, and *P*=.04, respectively) in processing quality by at least 1 out of the top 3 methods.

**Conclusions:**

These substantial participation numbers, large citation counts, and significant performance improvements encourage continuing to develop these technologies to address patient needs. Consequently, data and tools have been opened for future research and development, and the CLEF eHealth initiative continues to run new challenges.

## Introduction

The requirement to assure that patients can understand their own care epicrises, discharge summaries, and other electronic health (eHealth) records are stipulated by policies and laws (Multimedia Appendix 1) [1]. For example, the Declaration on the Promotion of Patients’ Rights in Europein 1994 by the World Health Organization states that all patients have the right to be fully informed about their own health status, prognosis, medical conditions, diagnoses, proposed and alternative treatment with potential risks and benefits, effects of nontreatment, treatment progress, and discharge guidelines. It also obligates health care workers to give every patient a written summary of this information and communicate it in a way appropriate to the patient’s capacity for understanding, including minimal use of unfamiliar jargon.

**Figure 1:**
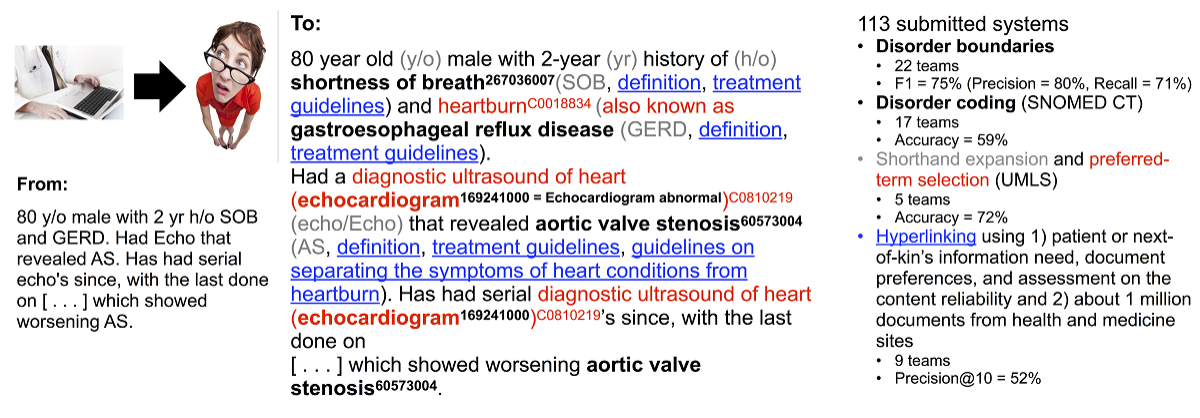
Original text, its enrichment, and submission statistics from the Conference and Labs of the Evaluation Forum (CLEF) eHealth 2013 evaluation lab. The year of 2013 has been chosen as an example here to illustrate the outcomes of the first year of organizing CLEF eHealth evaluation lab. SNOMED CT: Systematized Nomenclature of Medicine–Clinical Term, UMLS: Unified Medical Language System.

However, patients, their next of kin, and other laypersons are likely to experience difficulties in understanding the arcane jargon of eHealth records, and improving this readability can contribute to patient empowerment [2], defined as providing partial control and mastery over health and care which leads to patients having an active role in their health care, making better health/care decisions, being more independent from health care services, and having decreased costs of care [3]. This could mean replacing jargon words with patient-friendly synonyms, expanding shorthand, and providing an option to see the original text (Figure 1). Medical Subject Headings (MeSH), Systematized Nomenclature of Medicine–Clinical Terms (SNOMED CT), Unified Medical Language System (UMLS), and other terminology standards can help to define synonym replacements, but automated language processing is needed to identify text snippets to be replaced with synonymous snippets.

Patient-friendly language in health records can help patients make informed decisions, but this also depends on their access to consumer leaflets and other further supportive information about their health concerns. The internet is a powerful source for this information; most people will turn to its large range of content that is widely accessible and searchable [4,5]. However, layperson searches for medical information online can lead to the escalation of concerns and consequent anxiety [6]. Hence, helping patients retrieve relevant, understandable, and reliable information on the internet is crucial.

Web-based eHealth records provide a way to bridge patients’ actions of reading their own eHealth records with them searching the internet for further information. These eHealth records are targeted to both patients and health care workers for reading, writing, and sharing information [7]. Combined with the aforementioned record processing, this could mean enriching the health record with hyperlinks to term definitions, care guidelines, and other information on patient-friendly and reliable sites on the internet (Figure 1) as one way to facilitate patients in understanding their health and health care [2].

This paper reports on the 6 installations of CLEF eHealth, organized as part of the Conference and Labs of the Evaluation Forum (CLEF) initiative from 2012 to 2017. In 2012, it ran as a scientific workshop with the aim of establishing an evaluation lab, and since 2013, this annual workshop has been supplemented with 3 or more preceding labs each year. An evaluation lab is an activity where the participating individuals or teams’ goal is to solve the same problem, typically using the same dataset in a given time frame. In the CLEF eHealth 2013 to 2017 installations, the aim was to address patient-centric text processing. From 2015, the scope was also extended to aid both patients’ understanding and clinicians’ authoring of various types of medical content. CLEF eHealth 2017 introduced a new pilot task on technology-assisted reviews (TARs) in empirical medicine in order to support health scientists and health care policymakers’ information access.

Our focus in this article is on measuring and analyzing the scholarly influence of CLEF eHealth from 2012 to 2017. Its citation analysis, problem specifications, evaluation methods, data releases, software releases and submissions, and participation and benchmark results are addressed.

## Methods

The scholarly influence of the CLEF eHealth installations from 2012 to 2017 was measured by conducting a bibliometric study—an established method to provide a quantitative and qualitative indication of scientific activities whose use is also emerging in the context of evaluation initiatives [8-10]—of the related publications and their citations received by October 31, 2017. This study consisted of publication data collection, citation data collection, and data analysis.

The first 2 out of these 3 standard steps were concerned with the collection of materials for the measurement. First, conference paper and working note (ie, technical reports) publication data relevant to CLEF eHealth was collected from the CLEF proceedings (see Multimedia Appendix 2). These were supplemented with author-declared papers that extend these publications or otherwise use the CLEF eHealth datasets. Then, citation data for the resulting publication data were collected on October 26, 2017, from Google Scholar, one of the most comprehensive citation data sources in general and in particular for computer science, which is the main field of many CLEF eHealth scientists.

The third step formed the method of the study. Namely, citation content analysis [11], founded on content analysis [12] and grounded theory (introduced in the 1960s) [13], was used for the data analysis. This allowed a systematic, replicable compression of materials from the first 2 steps as codes and testing of hypotheses about the quantity and quality of the scholarly influence of CLEF eHealth from 2012 to 2017. Citation content analysis was chosen over the more established content analysis (Google Scholar had over 15,000 citations for the aforementioned paper [12] about this method) and grounded theory (Google Scholar had nearly 400 citations of the 2007 revision [13] of grounded theory for health sciences) because it combined these 2 research techniques for interpreting meaning from the content of text data as 1 overarching method for content coding of scientific literature and analysis of the codified content.

Using Google Scholar for citation data collection in bibliometric studies had at least 2 shortcomings [14-16]: first, paper duplication as a citation entry was frequent, for example, due to misspellings or incorrectly identified years and would, without manual refinement, cause errors in the counts. Another source for counting errors was incorrect automated merging of citation entries because of the same or almost the same title of a given conference paper and its journal extension. Similar to the scholarly influence measurement of CLEF 2000–2009 [9], our citation counts by Google Scholar were reviewed and refined for these 2 shortcomings by hand.

As part of the citation content analysis, the included publication and citation data were codified for 10 content categories: participation (including both expression of interest [EOI] and submission), author, affiliation, problem specification, evaluation method, benchmark result, data release, software launch, demonstration system, and citation. Similar to the bibliometric study [9], attention was paid not only to the number of citations but also the number of authors, their affiliations, and countries of affiliation. In order to illustrate the influence to the scholarly community and the individual scholars (because most participating teams included graduate students and/or early career academics), the scholarly influence was computed by multiplying the number of citations (ie, 1299, also known as scholarly impact [8-10]) for the included 184 papers by the number of their coauthors (ie, 741).

## Results

### Citation Analysis From 2012 to 2017

**Figure 2:**
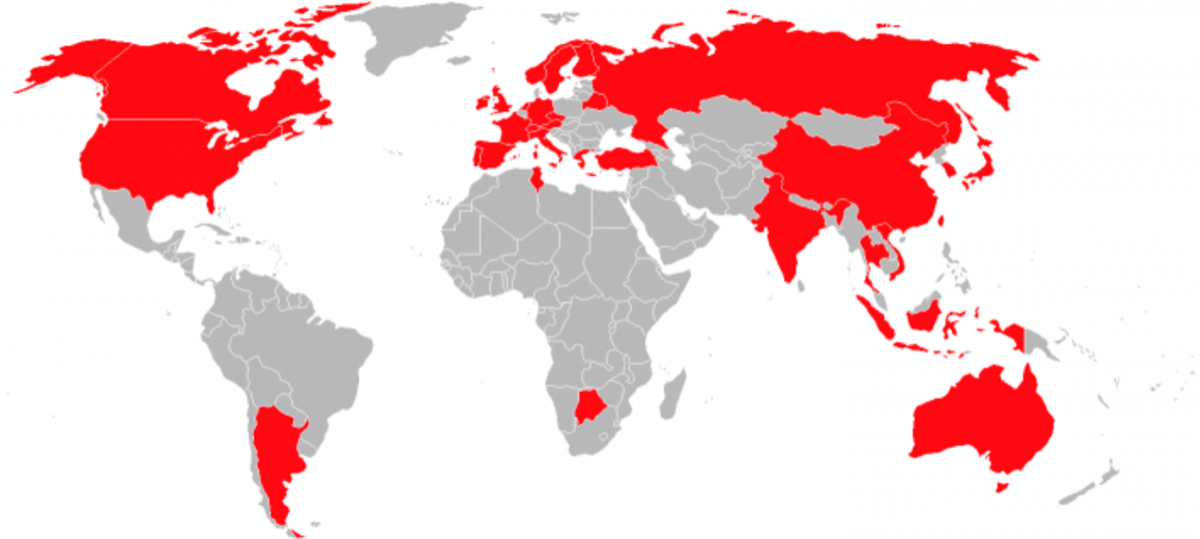
Map of the Conference and Labs of the Evaluation Forum (CLEF) eHealth 2012 to 2017 authors’ affiliation countries in red.

The topic of patient-friendly multilingual communication formed the focus of CLEF eHealth from 2012 to 2017 and generated a total scholarly influence of 962,559 citations (and scholarly impact of 1299 citations) for the 184 CLEF eHealth papers and reached 741 authors from 33 countries across the world (Multimedia Appendix 3, Figure 2) [17-22]. Of the 184 papers, 143 (77.7%) had been cited at least once and the maximum, mean, median, and standard deviation of citations per paper were 147, 7, 3, and 15, respectively. The h-index (ie, the number of papers each of which with at least h citations) and i10-index (ie, the number of papers with at least 10 citations) were 18 and 35, respectively. The annual number of published papers was 16, 35, 34, 31, 33, and 35 in 2012, 2013, 2014, 2015, 2016, and 2017, respectively. Although a clear 158 majority of the 184 papers were working notes (85.9%), 22 conference papers (12.0%) and 4 journal papers (2.0%) were also published.

In accordance with the CLEF eHealth mission to foster teamwork, the number of coauthors per paper was 4 on average, with a maximum, median, minimum, and standard deviation of 15, 3, 1, and 3, respectively. In 47 out of the 184 papers (25.5%), this coauthoring collaboration was international and sometimes even across continents (ie, Africa–Europe, Asia–Australia, Asia–Europe, Asia–North America, Australia–Europe, Australia–Europe–North America, and Europe–South America). Of the 466 author organizations, 427 (91.6%) were academic; 21 (4.9%) government and 18 (4.2%) industry organizations participated from 2012 to 2017.

CLEF eHealth particularly welcomed and attracted multidisciplinary teams to collaborate and bridge the researchers, scientists, lecturers, and graduate students with engineers, practitioners, and policy makers. For example, the 33 working notes and 1 conference paper from the CLEF eHealth 2013 evaluation lab [18] included 162 authors from 10 countries and featured some leading organizations in health information management, extraction, and retrieval, including National Information and Communications Technology Australia (NICTA), Commonwealth Scientific and Industrial Research Organization, and Health Language Laboratories from Australia; Chinese Canon Information Technology; French National Center for Scientific Research; Indian RelAgent Private Lt; US National Center for Biotechnology Information, Kaiser Permanente, and Mayo Clinic; and universities from Australia, China, Finland, Ireland, Republic of Korea, Spain, Sweden, United Kingdom, and United States. They represented academic, government, and industrial research labs, large technology corporations and smaller businesses, and health care providers and insurers.

### Problem Specifications From 2013 to 2017

The first installations of the lab, held in 2013 and 2014, focused on text processing, search, and visualization to ease patients’ (or their next of kin) understanding of hospital discharge summaries. Each year, 3 tasks were organized.

The 2013 tasks 1a and 1b considered disorder naming (eg, heartburn as opposed to gastroesophageal reflux disease) by identification of disorder names and normalization of the identified names by translating them to patient-friendly synonyms. These tasks could be illustrated as follows: the system should first automatically recognize GERD as a named entity in the phrase “80 y/o male with 2 yr h/o GERD,” associated with the entity code in SNOMED CT. Then, it should map the code to its most patient-friendly entry term, being heartburn as opposed to gastroesophageal reflux disease in the example phrase.

The 2013 task 2 on shorthand expansion aimed at mapping clinical abbreviations and acronyms to patient-friendly synonyms (eg, automatically expanding and mapping the 3 italicized text snippets in “80 y/o male with 2 yr *h/o SOB* and *GERD*” to history of, shortness of breath, and heartburn, respectively). Instead of actually writing the disorder names and shorthand expansions in the 2013 tasks 1b and 2, the respective SNOMED CT and UMLS codes (eg, GERD got the SNOMED CT code C0017168 in task 1b and UMLS code C0018834 in task 2) were applied. These coding systems were chosen because they are among the most commonly used in clinical settings.

This challenge continued in the 2014 task 2 on template filling, with the aim of developing attribute classifiers that predict the values of the UMLS concept unique identifiers (CUIs) with mention boundaries. The disease/disorder templates consisted of the following 10 attributes: negation indicator, subject class, uncertainty indicator, course class, severity class, conditional class, generic class, body location, DocTime class, and temporal expression.

The 2013 task 3, 2014 task 3, and 2014 task 1 supplemented the processing of health records with information from the internet, based on the patient’s (and next of kin’s) information needs associated with the records. The 2013 and 2014 task 3 on information search (information retrieval [IR]) would, for example, find the definition of shortness of breath, treatment guidelines for heartburn, and guidelines on separating the symptoms of heart conditions from heartburn for the health record with the aforementioned sentence. The challenge also considered in 2014 the problem of an individual expressing their information need in a non-English language, for search on Web pages written in English. Support of this functionality is important given the large proportion of Web medical content written in English. The 2014 task 1 on interactive information visualization had the overall goal of designing an effective, usable, and trustworthy environment for navigating, exploring, and interpreting health information as needed to promote understanding and informed decision making. It was divided into 2 parts as linkages to the three 2013 tasks, with tasks 1 and 2 on text classification as the first part and task 3 on IR as the second part. The scenario of the 2014 task 1 was an English-speaking, discharged patient (or next of kin) in their home in the United States. By reading their discharge document and further information on the internet on either a networked desktop system or mobile device (eg, mobile phone or tablet), they wanted to learn about their own health and health care in general and clinical treatment history, current symptoms and developments, and future implications in particular.

In 2015 and 2016, CLEF eHealth expanded its scope to multilingual text processing, medical Web search, and speech-to-text conversion to ease both patients (and their next of kin) and clinicians’ understanding of various types of medical content. Again, 3 tasks per year were organized.

The 2015 and 2016 task 1 built on processing tasks, data, and software by considering its nursing handover report support [23]. In clinical handover between nurses, verbal handover and note-taking could lead to loss of information, and electronic documentation was seen as laborious, taking time away from patient education. The challenges addressed taking clinical notes automatically by using speech recognition (SR) to convert spoken nursing handover into digital text and using information extraction (IE) to fill out a handover form.

The 2015 and 2016 task 2 considered clinical named entity recognition on French texts, previously an unexplored language. They aimed to automatically identify clinically relevant entities from French biomedical articles. In addition, the 2016 task also addressed extracting causes of death from French death reports.

The 2015 and 2016 task 3 considered cross-lingual medical search on the Web. They focused on trying to retrieve relevant and reliable Web pages that meet a given patient’s (or their next of kin’s) general information needs related to their medical complaints (eg, their need to understand a condition or the cause of a medical symptom). The tasks also considered information needs that were expressed in several non-English languages.

In 2017, the following 3 tasks were organized to continue the 2016 tasks 2 and 3 and introduce a new pilot task: 2017 task 1 explored the problem of multilingual text processing by considering the extraction of causes of death from both French and English death reports to ease clinicians’ understanding of these reports. The 2017 task 3 developed medical Web search techniques to address the challenge posed by patients (or their next of kin) in locating relevant and reliable medical content on the Web. In addition, the 2017 task 2 considered a new challenge, that of TAR generation in empirical medicine to support health care and policy making. Medical researchers and policy makers, while writing systematic review articles (eg, covering the treatment of a condition), must ensure that they consider all documents relevant to their review. As the size of medical libraries continues to expand, automation in this process is necessary.

### Evaluation Methods From 2013 to 2017

The evaluation criterion in the 2013 task 1a on disorder identification was the correctness in identification of disorder text snippets as defined by the F1 measure with a nonparametric test called random shuffling for the statistical significance assessment on 100 annotated health records for testing. An independent set of 200 annotated health records was provided for training. When computing true positives for the exact F1, the snippets by the solution-system and hand-annotation had to be identical, while an overlap was enough for the relaxed F1.

The evaluation criterion in the 2013 task 1b on disorder normalization was the correctness in mapping the disorders to SNOMED CT codes as defined by the accuracy measure with random shuffling for the statistical significance assessment. The annotated health records and their split between training and testing were the same as in task 1a. When computing true positives for the exact accuracy, the total number of code mappings was computed from the annotated records and the system was penalized for missing codes the same way as for incorrect codes. For the relaxed accuracy, the system was only evaluated on annotations that were detected by the system—that is, the total number corresponds to the code mappings with strictly correct text snippet generated by the system.

The evaluation criterion in the 2013 task 2 on shorthand extension was the correctness in mapping the preidentified shorthand to UMLS codes. This criterion was formalized using the exact and relaxed accuracy measures with random shuffling for the statistical significance assessment. The annotated health records and their split between training and testing were the same as in task 1a.

Evaluation of submissions to the 2013 task 3 on IR was conducted with respect to the relevance of the retrieved documents to the information seeker on 50 test queries and the matching result set. The official primary and secondary measures were the precision at 10 (P@10) and normalized discounted cumulative gain at 10 (NDCG@10), respectively. The Wilcoxon test was used to better compare the measure values for the runs and benchmark.

In the 2014 task 1 on information visualization, participants could submit their designs to an optional draft submission to receive comments, followed by the call for final submissions. Final submissions were judged on their rationale for the design, including selection of appropriate visual interactive data representations and reference to state-of-the-art techniques by an expert panel with 5 members. To be successful, the submission had to demonstrate that the posed problems and information needs are addressed, provide a compelling use-case driven discussion of the work flow supported and exemplary results obtained, and highlight the evaluation approach and obtained findings. Primary judging criteria included the effectiveness and originality of the proposed design that were further divided to categories for aesthetics, interaction, usability, and visualization.

Evaluations in the 2014 tasks 2 and 3 followed the 2013 practices. In the 2014 task 2 on template filling, exact and relaxed versions of accuracy and F1 were used. In the 2014 task 3 on IR, participants were provided with 50 topics, including 5 training topics, with their translation in Czech, German, and French. Primary and secondary evaluation measures were P@10 and NDCG@10, respectively.

The 2015 task 1 on speech recognition evaluation used error in speech recognized words, and 100 training and 100 test documents were provided.

The 2015 task 2 on named entity recognition had 3 subtasks that were evaluated separately: (1) for plain entity recognition, raw text was supplied to participants who had to submit entity annotations comprising entity offsets and entity types, (2) for normalized entity recognition, raw text was supplied to participants who had to submit entity annotations comprising entity offsets, entity types, and entity normalization (UMLS CUIs), and (3) for entity normalization, raw text and plain entity annotations were supplied to participants who had to submit entity normalization (UMLS CUIs). For each of the subtasks, the system output on the unseen test set was compared to the gold standard annotations, and precision, recall, and F1 were computed.

In 2015 task 3 on IR, evaluation was conducted using similar measures as previous years: P@10 and NDCG@10 were the primary and secondary measures, respectively. A separate evaluation was conducted using both relevance assessments and readability assessments. For all runs, rank-biased precision was computed along with its readability-biased modifications for the binary readability assessments and the graded readability assessments.

In 2016, the nursing handover support task used precision, recall, and F1 for evaluation. Performance was evaluated first separately in every heading from 1 to 35 and the 36th heading for irrelevant text. Then, the performance was averaged over the 35 form headings and also documented in the dominant class of 36. The Wilcoxon test was used for statistical significance testing. The previous 200 training and test documents were provided for training; they were supplemented by another 100 documents for testing.

For the 2016 task 2 and 2017 task 1 on IE, the system output on the unseen test set was compared to the gold standard annotations, and precision, recall, and F1 were compared. After submitting their result files, participating teams had 1 extra week to submit the system used to produce them or a remote access to the system, along with instructions on how to install and operate the system for the replicability to be tested.

In 2016 and 2017, for the IR task, evaluation was conducted using P@10 and NDCG@10 as the primary and secondary measures, respectively. Precision was computed using the binary relevance assessments; NDCG was computed using the graded relevance assessments. A separate evaluation was conducted using the multidimensional relevance assessments (topical relevance, readability, and trustworthiness). For all runs, rank-biased precision was computed along with its multidimensional modifications for the binary readability assessments, the graded readability assessments, and the binary readability and trustworthiness assessments. In 2017, these measures were parameterized for a given user’s expertise.

In the 2017 pilot task on TAR in empirical medicine, evaluation measures were area under the recall-precision curve, minimum number of documents returned to retrieve all relevant documents, work saved over sampling at different recall levels, area under the cumulative recall curve normalized by the optimal area, recall @ 0% to 100% of documents shown, a number of newly constructed cost-based measures, and reliability.

### Data Releases From 2013 to 2017

The CLEF eHealth 2013 tasks used the 300 deidentified, manually annotated (for disorder names and clinical shorthand) health records of the Shared Annotated Resources (ShARe) corpus of the Multiparameter Intelligent Monitoring in Intensive Care (MIMIC) II database, consisting of discharge summaries and electrocardiogram, echocardiogram, and radiology reports.

To enable IR, 55 new search topics were formed specifically for task 3. Each search task was described using a patient profile (eg, a 40-year-old woman, who seeks information about her condition), information need (eg, description of what type of disease hypothyroidism is), and query with separate fields for its title (eg, Hypothyroidism) and description (eg, What is hypothyroidism?). The profile also allowed the participants to address the task without considering the aforementioned health records. To create result document sets for these search tasks, a large crawl of online health resources targeted to laypeople and clinicians and provided by the Knowledge Helper for Medical and Other Information users (Khresmoi) project was used.

The CLEF eHealth 2014 task 1 built on these 2013 datasets by combining them as a whole in order to address information search and visualization in a patient-centric way. One mandatory and 5 optional patient cases were carefully chosen from the 2013 tasks 1 to 3 for this task [24]. These consisted of search topics and result sets from task 3 and associated annotated discharge summaries from tasks 1 and 2.

The 2014 task 2 on template filling also used the 2013 dataset of 300 deidentified health records, supplemented by a test set of 133 unseen discharge documents and new expert annotations created as part of the ShARe project. The annotations extended the existing disorder annotations from the 2013 task 1 by focusing on template filling for 10 different attributes for each disorder mention.

To enable IR in the 2014 task 3, 55 new queries were first formulated by experts from the main disorders diagnosed in discharge summaries provided in the 2014 task 2 and then associated with result document sets of the aforementioned Khresmoi set. Participants were provided with the mapping between queries and discharge summaries and were again given an option to use the discharge summaries.

The CLEF eHealth 2015 and 2016 targeted 2 new tasks as its tasks 1 and 2, in addition to continuing its established and popular series of IR tasks as its task 3. The new task 1 focused on supporting handover communication with 300 synthetic patient cases for the SR training, validation, and testing in 2015 and IE training, validation, and testing in the 2016 task 1. Each case in this NICTA Synthetic Nursing Handover Data consisted of a patient profile; a written, free-form text paragraph (ie, the written handover document) to be used as a reference standard in SR; and its spoken (ie, the verbal handover document) and speech-recognized counterparts. The written handover documents were annotated by a registered nurse using a form with 49 headings (ie, classes) to fill out.

For the new 2015 and 2016 task 2, two types of biomedical documents were used: a total of 1668 titles of scientific articles indexed in the MEDLINE database and 6 full-text drug monographs published by the European Medicines Agency. These were annotated with 10 types of entities of clinical interest defined by semantic groups in the UMLS. The expert annotations marked each relevant entity mention in the documents and assigned the corresponding semantic types and CUIs. The 2016 task 2 also featured a subtask that used the CépiDC Causes of Death Corpus with free-text descriptions of causes of death as reported by physicians in the standardized causes of death forms. Each document (65,843 death certificates in total) was manually annotated by experts with the codes from the *International Statistical Classification of Diseases and Related Health Problems, Tenth Revision* (ICD-10) per the international World Health Organization standards. Manually built dictionaries of terms associated with the annotated ICD-10 codes were also released.

The 2015 task 3 considered the following scenario to generate 67 English queries: a patient or their next of kin is first shown images and videos related to medical symptoms and then asked which queries they would issue to a Web search engine if they were exhibiting such symptoms and wanted to find more information to understand these symptoms or their condition. In 2016, 6 queries were generated for each information need by having individuals with different levels of medical expertise formulate queries based on the content of posts extracted from the askDocs section of the Reddit public health Web forum. For the multilingual query set, queries were translated by experts to Arabic, Czech, German, Farsi, French, Italian, and Portuguese in 2015 and Czech, German, French, Hungarian, Polish, and Swedish in 2016. The Khresmoi document collection was used in 2015, and a new document collection, ClueWeb12 B13, in 2016. Along with relevance assessments by expert assessors on the result document sets, readability judgements were also collected for the assessment pool in 2015 and both readability and reliability in 2016.

Finally, in 2017, the CLEF eHealth 2016 tasks 1 and 3 were extended and the aforementioned new pilot task with unseen data was introduced as the CLEF eHealth 2017 task 2. The 2017 task 1 used a corpus of expert-annotated death certificates from France in French and the United States in English with respect to the ICD-10 codes. Again, this task supplemented its data releases by manually built dictionaries of terms associated with the annotated ICD-10 codes. The 2017 task 3 used the same document collection and topics as in 2016, with the aim of acquiring more relevance assessments and improving the collection reusability.

The new TARs in empirical medicine task (ie, the 2017 task 2) used a subset of MEDLINE documents for its challenge to make abstract and title screening more effective. The PubMed identifiers (PMIDs) of potentially relevant MEDLINE document abstracts indexed by the PubMed search engine were provided for 20 training and 30 test topics. The PMIDs were collected by the task coordinators by rerunning the MEDLINE Boolean query used in the original systematic reviews conducted by Cochrane to search PubMed. Topics consisted of the Boolean search from the first step of the systematic review process: a topic identifier; title of the review, written by Cochrane experts; Boolean query manually constructed by Cochrane experts; and set of PMIDs returned by running the query in MEDLINE. The original systematic reviews written by Cochrane experts included a reference section that listed included, excluded, and additional references to medical studies. The union of included and excluded references were the studies that were screened at a title and abstract level and considered for further examination at a full content level. These constituted the relevant documents at the abstract level, while the included references constituted the relevant documents at the full content level. References in the original systematic reviews were collected from a variety of resources, not only MEDLINE. Therefore, studies that were cited but did not appear in the results of the Boolean query were excluded from the label set.

### Software Releases and Submissions From 2013 to 2017

CLEF eHealth began providing participants with software and code for method evaluation, record text annotation, and document relevance assessment in 2013 and extended this to also release processing code in 2016. The software and code releases were motivated by our desire for faster progress, comprehensive benchmarking, and transparency of the CLEF eHealth outcomes. Prior to CLEF eHealth, the progress in eHealth information and communication technology (ICT) was extremely limited in comparison to banking, defense, and many other fields that also record big data and benefit from their analytics because of barriers in limited collaboration in sharing data, processing methods, and evaluation outcomes together with their common conventions and standards [25].

In the CLEF eHealth 2013 tasks 1 and 2, we released both a command-line tool and a graphical user interface that the participants could use to compute the values for the official and supplementary evaluation measures and visualize annotations against their method outputs. This eHOST annotation tool [26] also supported participants in annotating more data, although methods using teams’ own annotations were evaluated separately from those based on the organizers’ original annotations alone. In the CLEF eHealth 2013 task 3, we released the Relevation! relevance assessment tool [27] and provided participants with a pointer to an established tool for computing values for the official and supplementary evaluation measures.

The 12 CLEF eHealth 2014 to 2017 tasks in total continued releasing software and code for computing values for evaluation measures, evaluating statistical significance of their differences between 2 or more methods, data annotation, and relevance assessment. In addition to releasing purpose-built software and code for the tasks, pointers to such helpful resources by other tasks and groups were also catalogued and provided on the website and overview paper of each task.

The CLEF eHealth 2016 task 1 released the organizers’ entire software stack as a state-of-the-art solution to the handover IE problem (ie, both feature generation and IE) [23]. Participants were welcomed but not mandated to use the released code and, as intended, the results highlighted all participating teams’ methods outperforming this known state-of-the-art baseline.

In parallel to these software and code releases, CLEF eHealth established its replication track in 2016. The track gave the participants of the 2016 task 2 and 2017 task 1 the opportunity to submit their processing methods to organizers, who then attempted to replicate the run submissions. In 2016, 3 participating teams chose this option and submitted a total of 7 methods, all of which the organizers were able to replicate perfectly. In 2017, 5 participating teams chose the replication track and submitted a total of 22 methods. The organizers were able to replicate most of them perfectly without contacting the teams. Where team contact was required, replication was achievable after further technical clarification on system requirements, installation procedure, and practical use. The organizers also reported an overall improvement in method documentation as an outcome of running the track twice.

### Participation and Benchmark Results From 2013 to 2017

The CLEF eHealth lab each year from 2013 to 2017 attracted more than 100 teams to submit their EOI for the task and among them, 20 to 34 teams participated (Multimedia Appendix 3). The difference between the number of teams interested and the actual participation was explained by the ease of the registration process versus the substantial amount of work required to actually submit to these difficult tasks. The very high number of EOIs within the first 2 years was surely related to the novelty of the 2013 and 2014 tasks. The number of participants from 2013 to 2017 remained stable over the years despite the regular change and diversity in tasks. The most popular tasks were related to the IR task 3 in 2013 to 2017. Given that both the number of EOIs and participants have decreased for the last 2 years, the task might have to be redefined.

The results of the 15 tasks organized as part of the CLEF eHealth lab from 2013 to 2017 contributed to the body of knowledge about the difficulty of health information management, extraction, and retrieval (Multimedia Appendix 4). In addition, the methodological diversity of the submissions shown by more than 100 teams all over the world, together with the baselines by the organizers, addressed the applicability of particular methods. Eight tasks produced statistically significant improvements in processing quality by at least 1 of the top 3 methods.

## Discussion

### Principal Findings

The CLEF eHealth installations have offered 15 evaluation labs in the fields of medical information management, extraction, and retrieval since 2012. Evaluation methods and resources have been developed and shared with the community to support the understanding of and access to medical content by laypeople (or their next of kin), clinicians, scientists, and policy makers. Evaluation results for the methods and resources developed have been released to the community. In doing so the lab has provided an evaluation setting for the progression of research in multilingual medical ICT. This has facilitated further evaluation into medical system development for information management, extraction, and retrieval and aiding the progression of research in these areas.

The annual CLEF eHealth lab workshop held at the main CLEF conference provides for the dissemination and discussion of the outcomes of each year’s challenges. This has facilitated discussion in the community, cross-fertilization of ideas, and further progress in the medical information production, processing, and consuming ecosystem. Each year the lab organizers produce lab overview papers describing the challenges offered and participants’ results. These have proven influential, as indicated by their citation indexes.

### Comparison With Prior Work

At least 12 years prior to establishing CLEF eHealth in 2012, evaluation labs began addressing limited collaboration as a major barrier that hinders the transfer of ICT for processing free-form text to clinical practice, and this is evidenced by improvements in developing and sharing data, community conventions, standards, software, and evaluation benchmarks [25]. The other 2 identified main barriers were absence of user centricity in technology development and inabilities to replicate results. By definition as a lab, CLEF eHealth 2012–2017 continued contributing to the barrier of limited collaboration but used the remaining 2 barriers to distinguish itself from other labs. Namely, it placed layperson patients (as opposed to clinical experts) as targeted technology users to the center of the shared tasks in 2013 and introduced its replication track in 2016.

The CLEF initiative began in Europe in 2000, and at the same time that the first CLEF eHealth evaluation lab with 3 shared tasks was launched in 2013, the CLEF Question Answering for Machine Reading lab introduced a pilot task on machine reading on biomedical text about Alzheimer disease [28]. Extending the prior work inclusion criterion from text to other data modalities, the ImageCLEF lab included annual shared tasks on biomedical image processing from 2005 to 2013 [29-31].

Before CLEF, the Text Retrieval Conference (TREC) was established in the United States in 1992 as an evaluation initiative with evaluation labs of shared tasks leading to annual conferences and workshops. In 2000, the TREC filtering tasks considered user profiling to filter in only the topically relevant biomedical abstracts using MeSH as topics [32]. From 2003 to 2007, the TREC genomics tasks ranged from ad hoc IR to text classification, passage retrieval, and entity-based question answering on data from biomedical papers and eHealth records [33]. In 2011 and 2012, the TREC medical records tasks targeted building a search engine where the patient cohort’s eligibility criteria of a given clinical study can be specified through the search query, and then after information search on English eHealth records, the matching population is returned for study recruitment purposes [34]. This paper had 17 citations by July 6, 2018.

The NII Test Collection for Information Retrieval Systems was launched in Japan in 1997 as an evaluation initiative and in 2013, its medical natural language processing lab considered the following 3 shared tasks on Japanese eHealth records [35]: text deidentification, complaint/diagnosis IE, and an open challenge, where participants were given the freedom to try to solve any other task on the dataset that was used for the first 2 tasks. This paper gathered 33 citations by July 6, 2018.

The Informatics for Integrating Biology and the Bedside initiative, begun in the United States in 2006, addressed clinical text processing through its following shared tasks on English-language eHealth records from 2006 to 2012 [36]: text deidentification and identification of smoking status in 2006; recognition of obesity and its comorbidities in 2008; medication IE in 2009; concept, assertion, and relation recognition in 2010; co-reference analysis in 2011; and temporal-relation analysis in 2012. This paper had 491 citations by July 6, 2018.

The Medical Natural Language Processing Challenges, launched in the United States in 2007, considered automated diagnosis coding of English-language radiology reports from a children’s radiology department in 2007 and classifying the emotions found in English-language suicide notes in 2011 [37,38]. These papers were cited in total 603 times by July 6, 2018.

The annual SemEval/Senseval Workshops, established in 2004 to address semantic disambiguation, role labelling, IE, IR, frame extraction, temporal annotation, and other multilingual semantic processing tasks, adopted our CLEF eHealth data in 2014 [39]. By supplementing our annotations for the CLEF eHealth 2013 tasks 1 and 2, it challenged its participants to the same tasks but on a larger test set. A total of 21 participating teams completed this SemEval 2014 task 1 with the strict-F1 of 81.3% at its best; 18 of those teams also participated in the SemEval 2014 task 2 with the top strict-accuracy of 74.1%. The citation count of this paper was 71 by July 6, 2018.

### Limitations

In this paper, we have presented a bibliometric study of the scholarly influence of CLEF eHealth installations from 2012 to 2017. The paper and citation data collection have been limited to the CLEF eHealth proceedings and previously catalogued papers and were conducted only 2 months after the CLEF eHealth 2017 proceedings were published. Consequently, other relevant papers and citations are likely to exist, making our citation influence of 1299 citations in total for the 184 papers by the 741 coauthors from 33 countries a modest rather than exaggerated estimate.

In comparison, the scholarly influence of 6 TREC video retrieval installations from 2002 to 2009 has been evaluated retrospectively 2 years after the last installation as 15,828 citations for the 2073 papers (of which 319 have been published in the TREC conference paper or working note proceedings) [8]. A comparable influence has been achieved within the CLEF initiative by its ImageCLEF activity from 2000 to 2009 [9]. First, 7 ImageCLEF installations were evaluated retrospectively in 2013 (4 years after the 2009 installation) as having had the influence of 2018 citations for the 179 papers. Second, the scholarly influence of 10 installations of the entire CLEF initiative from 2000 to 2009 has been evaluated retrospectively in 2013 (4 years after the 2009 installation) as 9137 for the 873 papers.

Our average number of citations generated by a paper (ie, 7) is smaller than this number is for the entire CLEF initiative (ie, 10) but larger than what many other subinitiatives achieved (from 0.2 to 35, with 11 for ImageCLEF) [9]. CLEF eHealth, established in 2012, is not included in this comparison of 16 CLEF subinitiatives with up to 10 installations each. Moreover, our numbers for 7 installations originate from the year of the last installation as opposed to being collected 4 years after.

Although the CLEF eHealth installations have attracted substantial community interest, as reflected by the 741 coauthors of the 184 papers from 33 countries, we do not really have sufficient participation from Africa, Central and South America, and the Middle East. However, this problem of insufficient participation has been acknowledged by a recent review of evaluation initiatives in biomedical text mining from 2002 to 2014 as one of the main conclusions [40]. Fortunately, we have been successful in targeting the coupled problem of insufficient innovation by reaching statistically significant improvements in most CLEF eHealth tasks.

### Significance and Future Work

The CLEF eHealth installations with 15 information management, extraction, and retrieval tasks in total uniquely target various layperson (or next of kin) information understanding and provision challenges in the medical domain (Multimedia Appendix 2). Coupled with this, it strives to drive research in the fields of clinician information processing, exchange, and understanding support. Finally, for the first time globally it targets challenges toward meeting the needs of policy makers for TAR generation in empirical medicine. In IE, the lab has targeted named entity recognition and normalization in clinical reports and named entity recognition, normalization, and classification in biomedical articles and in death reports. In information management, the lab has considered medical data visualization and nurses’ handover report management. Finally, in IR the target has been on patient-centered search, cross-lingual search, and technology-assisted reviewing.

The lab has attracted considerable and growing interest from the research community over the years: 34 unique teams participated in the 3 tasks in 2013, 24 in the 3 tasks in 2014, 20 in the 3 tasks in 2015, 20 in the 3 tasks in 2016, and 32 in the 3 tasks in 2017. While the lab has yet to become entirely global, it is already far reaching, attracting participants from 33 countries.

By virtue of the lab series over the first 6 years of its life, from 2012 to 2017 inclusive, we conjecture that CLEF eHealth has influenced progress by (1) bringing the research community together through the lab series to collaborate and discuss challenges associated with technique development in the biomedical and clinical information management, extraction, and retrieval spaces; (2) providing access to shared data, resources, processing methods, and evaluation settings for eHealth system research, development, and evaluation; and (3) offering reproducibility, scalability, and user-centricity. While it is difficult to accurately quantify such influence, the 1299 citations with influence of circa 963,000 generated by the lab in its first 6 years of existence are suggestive. Progress in the areas addressed by the lab has the potential to generate high impact not only on the research field but more generally on society, given the importance of health information access to support health care as well as empower people to manage their health.

### Conclusions

In today’s information overloaded society it is increasingly difficult to retrieve and digest valid and relevant electronic medical information to make health-centered decisions. The CLEF eHealth lab aims to support the development of techniques to ease this challenge. Over the years this lab series has expanded its original goal of supporting patients (or their next of kin) in understanding the jargon in their hospital discharge summary to consider a broader set of medical information needs of both patients (or their next of kin), clinicians, scientists, and policy makers. Related to these themes, challenges have been offered in a multilingual setting on the topics of medical information management, extraction, and retrieval. The 15 challenge tasks, from 2013 to 2017, have attracted much attention, as evidenced by the annual lab overview papers, participants’ working notes papers, and external papers using the lab resources, obtaining a combined total of 184 papers by 741 coauthors from 33 countries across the world with 1299 citations, totalling a citation influence of circa 963,000. Given the significance of the lab series, all test collections and resources associated with the lab challenges have been made available to the wider research community through the internet.

The lab has attracted many participants from across the globe since its conception 6 years ago. In total, 718 teams have registered their interest in the lab tasks, leading to 130 teams submitting to these tasks. Together we have influenced the progression of health text processing and medical IR research. As the lab further progresses, we envision its scope and reach extending even further.

## Appendix

Multimedia Appendix 1 Electronic health records and other health information.

Multimedia Appendix 2 Timeline of Conference and Labs of the Evaluation Forum eHealth and related conference proceedings and working notes.

Multimedia Appendix 3 Summary of the bibliometric analysis of Conference and Labs of the Evaluation Forum eHealth from 2013 to 2017.

Multimedia Appendix 4 Summary of the benchmark results for the Conference and Labs of the Evaluation Forum eHealth tasks from 2013 to 2017.

## Abbreviations

CLEF: Conference and Labs of the Evaluation Forum, formerly known as Cross-Language Evaluation Forum
CUI: concept unique identifier
EOI: expression of interest
ICD-10: International Statistical Classification of Diseases and Related Health Problems, Tenth Revision
ICT: information and communications technology
IE: information extraction
IR: information retrieval
Khresmoi: Knowledge Helper for Medical and Other Information Users
MeSH: Medical Subject Heading
MIMIC: Multiparameter Intelligent Monitoring in Intensive Care
NDCG@10: normalized discounted cumulative gain at 10
NICTA: National Information and Communications Technology Australia
NLP: natural language processing
P@10: precision at 10
PMID: PubMed identifier
ShARe: shared annotated resources
SNOMED CT: Systematized Nomenclature of Medicine–Clinical Terms
SR: speech recognition
TAR: technology-assisted reviews
TREC: Text Retrieval Conference
UMLS: unified medical language system
