# Supplementary material for: Scholarly Influence of the Conference and Labs of the Evaluation Forum eHealth Initiative: Review and Bibliometric Study of the 2012 to 2017 Outcomes
Source: JMIR Res Protoc. 2018 Jul 27;7(7):e10961. doi: 10.2196/10961 (PMC6086930; doi:10.2196/10961)
Supplement: Multimedia Appendix 1 [file resprot_v7i7e10961_app1.pdf]

Multimedia Appendix 1: Electronic Health Records and Other Health Information

Health information refers to all health-related content in all data formats, document types, information systems, publication media, and languages from all organisations, states, and countries. The privacy-sensitive, official part of health information consists of data recorded in healthcare services when describing a given patient’s health or healthcare (Figure A.1). The accessibility of this data is defined as limited (i.e., private or confidential information) and it is recorded either on paper in health records or electronically in *Electronic Health* (eHealth) records.

Some common synonyms or related terms of eHealth records include eHealth charts, data, documents, information, letters, notes, reports, and summaries. Typical ways of making the term more specific consist of detailing the record type (e.g., admit/admission document, case sheet, discharge document, or handover form), adding the recording method (e.g., computer based, computerised, digital, electronic, or paper based), and adding the information format (e.g., categorical, free/free-form text, numeric, structured, or textual). The health records term can be generalised over the healthcare professions by using the term clinical records instead of, for example, dental records, medical records, and nursing records for documents authored by dentists, medical doctors, and nurses, respectively.

MeSH 2012: parent term	
**Heading** ***Entry terms***	**Scope**
**Records as topic** *Forms as topic* *Personal identification systems* *Travel documents*	The commitment in writing, as authentic evidence, of something having legal importance. The concept includes certificates of birth, death, etc., as well as hospital, medical, and other institutional records.

MeSH 2012: parent term			Australian term in 2012	
**Heading** ***Entry terms***	**Scope**	**MeSH 2006 interpretatio n**	**Heading**	**Scope**
**Health records, personal** *Patient records, computerized* *Personal electronic health records* *Personal health records* *Personal medical records*	Longitudinal patient-maintained records of individual health history and tools that allow individual control of access.	The entry term of *Health records, personal* was considered as a synonym of the heading of **Medical Records**.	**Personally Controlled Electronic Health Record**	A secure, electronic record of a person's important health information, which brings key and frequently-used elements of the person's health information together in a unified record and is accessible only by the person and his/her authorised healthcare providers

MeSH 2012: children of Records as topic	
**Heading and entry terms**	**Scope**
Birth certificates Birth records	The commitment in writing, as authentic evidence, of something having legal importance. The concept includes certificates of birth, death, etc., as well as hospital, medical, and other institutional records.
Consent forms Consent documents Informed consent forms Informed consent documents	Documents describing a medical treatment or research project, including proposed procedures, risks, and alternatives, that are to be signed by an individual, or the individual's proxy, to indicate his/her understanding of the document and a willingness to undergo the treatment or to participate in the research
Death certificates Death records	Official records of individual deaths including the cause of death certified by a physician, and any other required identifying information
Dental records	Data collected during dental examination for the purpose of study, diagnosis, or treatment planning
Hospital records	Compilations of data on hospital activities and programs; excludes patient medical records
Medical records Health diaries Medical transcription	Recording of pertinent information concerning patient's illness or illnesses
Nursing records	Data recorded by nurses concerning the nursing care given to the patient, including judgment of the patient's progress, and other institutional records

Figure A.1. Illustration of terminological differences used to refer to health records in the Medical Subject Headings (MeSH), created and updated by the US National Library of Medicine from 2006 to 2012 and between MeSH and Australian terminology. The year 2012 has been chosen to reflect the linguistic landscape at the time of introducing CLEF eHealth in 2012.
