# Supplementary material for: Scholarly Influence of the Conference and Labs of the Evaluation Forum eHealth Initiative: Review and Bibliometric Study of the 2012 to 2017 Outcomes
Source: JMIR Res Protoc. 2018 Jul 27;7(7):e10961. doi: 10.2196/10961 (PMC6086930; doi:10.2196/10961)
Supplement: Multimedia Appendix 2 [file resprot_v7i7e10961_app2.pdf]

Multimedia Appendix 2: Timeline of CLEF eHealth and Related Conference Proceedings and Working Notes

Timeline of CLEF eHealth

In 2012, the CLEF initiative introduced eHealth with its workshop program including first three invited talks on collaborative data and software resources; oral talks for eleven papers; and a student mentoring session where PhD students presented their study plans, followed by feedback from their designated mentors. All these talks focused on meeting the needs of healthcare professionals and patients in ease of information recording, access, and understanding via user-centred abbreviation processing, content generation, search engines, and vocabularies, among other tools to support patient–professional interaction across languages and sub-languages. Then, the program continued to verifying this community interest in user-friendly multilingual communication through an expert panel, professional networking session, and a working session for developing a road map for CLEF eHealth 2013.

Since 2013, the annual CLEF eHealth workshop has been supplemented with three or more preceding labs each year with an aim to address patient-centric text processing (Table A.1). From 2015, the scope was also extended to ease both patients' understanding and clinicians' authoring of various types of medical content. CLEF eHealth 2017 also introduced a new pilot task on technology assisted reviews in empirical medicine in order to support health scientists and healthcare policymakers' information access.

The CLEF eHealth evaluation lab series runs for the seventh year in 2018. This CLEF eHealth 2018 edition of the lab continues the 2017 IE, TAR, and IR tasks. The 2018 IE task will extend to new European languages with new death reports for developing named entity recognition and normalisation. The 2018 TAR task will build on last year's task to offer a new evaluation framework with new evaluation measures and new data. Finally, the 2018 IR task will offer new queries and new evaluation criteria to support developing new techniques for faceted search and patient-centred IR. Beyond 2018, we envision CLEF eHealth growing further, to extend the scope of IE and IR related challenges offered, to offer new information management challenges, to increase multilingual approaches, and to extend the scope of the lab to consider other challenges relevant to biomedical, clinical, and eHealth content.

Table A.1. Timeline of CLEF eHealth Tasks. Abbreviations: *English* (EN), *French* (FR), *information retrieval* (IR), *technology assisted reviews* (TAR)

	**Task**	**Timeline [language, data type, task year]**				
		2013	***2014***	***2015***	***2016***	***2017***
**Information Extraction**	Named entity recognition and/or normalisation	EN clinical reports		FR biomedical articles		
	Extraction		EN clinical reports			Multilingual death reports
	Classification				FR death reports	
	Replication task				Code	
**Information Management**	Visualisation		EN eHealth data			
	Report generation and management			EN nursing handover reports		
**Information Retrieval (IR)**	Patient-centered IR	Multilingual eHealth data				
	Cross-lingual IR		Multilingual eHealth data			
	TAR in empirical medicine					EN biomedical articles

CLEF Conference Proceedings

Catarci T, Forner P, Hiemstra D, Penas A, Santucci G (eds) (2012) Information Access Evaluation. Multilinguality, Multimodality, and Visual Analytics. Proceedings of the Third International Conference of the CLEF Initiative (CLEF 2012), Lecture Notes in Computer Science (LNCS) 7488, Springer, Heidelberg, Germany. DOI:10.1007/978-3-642-33247-0

Forner P, Müller H, Paredes R, Rosso P, Stein B (eds) (2013a) Information Access Evaluation meets Multilinguality, Multimodality, and Visualization. Proceedings of the Fourth International Conference of the CLEF Initiative (CLEF 2013), Lecture Notes in Computer Science (LNCS) 8138, Springer, Heidelberg, Germany. DOI:10.1007/978-3-642-40802-1

Kanoulas E, Lupu M, Clough P, Sanderson M, Hall M, Hanbury A, Toms E (eds) (2014) Information Access Evaluation – Multilinguality, Multimodality, and Interaction. Proceedings of the Fifth International Conference of the CLEF Initiative (CLEF 2014), Lecture Notes in Computer Science (LNCS) 8685, Springer, Heidelberg, Germany. DOI:10.1007/978-3-319-11382-1

Mothe J, Savoy J, Kamps J, Pinel-Sauvagnat K, Jones GJF, SanJuan E, Cappellato L, Ferro N (eds) (2015) Experimental IR Meets Multilinguality, Multimodality, and Interaction. Proceedings of the Sixth International Conference of the CLEF Association (CLEF 2015), Lecture Notes in Computer Science (LNCS) 9283, Springer, Heidelberg, Germany. DOI:10.1007/978-3-319-24027-5

Fuhr N, Quaresma P, Goncalves T, Larsen B, Balog K, Macdonald C, Cappellato L, Ferro N (eds) (2016) Experimental IR Meets Multilinguality, Multimodality, and Interaction. Proceedings of the Seventh International Conference of the CLEF Association (CLEF 2016), Lecture Notes in Computer Science (LNCS) 9822, Springer, Heidelberg, Germany. DOI:10.1007/978-3-319-44564-9

Jones GJF, Lawless S, Gonzalo J, Kelly L, Goeuriot L, Mandl T, Cappellato L, Ferro N (eds) (2017) Experimental IR Meets Multilinguality, Multimodality, and Interaction. Proceedings of the Eighth International Conference of the CLEF Association (CLEF 2017), Lecture Notes in Computer Science (LNCS) 10456, Springer, Heidelberg, Germany. DOI:10.1007/978-3-319-65813-1

CLEF Proceedings for Working Notes:

Forner P, Karlgren J, Womser-Hacker C, Ferro N (eds) (2012) CLEF 2012 Working Notes, CEUR Workshop Proceedings 1178. ISSN: 1613-0073.

Forner P, Navigli R, Tufis D, Ferro N (eds) (2013b) CLEF 2013 Working Notes, CEUR Workshop Proceedings 1179. ISSN 1613-0073.

Cappellato L, Ferro N, Halvey M, Kraaij W (eds) (2014) CLEF 2014 Working Notes, CEUR Workshop Proceedings 1180. ISSN: 1613-0073.

Cappellato L, Ferro N, Jones GJF, SanJuan E (eds) (2015) CLEF 2015 Working Notes, CEUR Workshop Proceedings 1391. ISSN: 1613-0073.

Balog K, Cappellato L, Ferro N, Macdonald C (eds) (2016) CLEF 2016 Working Notes, CEUR Workshop Proceedings 1609. ISSN: 1613-0073.

Cappellato L, Ferro N, Goeuriot L, Mandl T (eds) (2017) CLEF 2017 Working Notes, CEUR Workshop Proceedings 1866. ISSN: 1613-0073.
