# Supplementary material for: Scholarly Influence of the Conference and Labs of the Evaluation Forum eHealth Initiative: Review and Bibliometric Study of the 2012 to 2017 Outcomes
Source: JMIR Res Protoc. 2018 Jul 27;7(7):e10961. doi: 10.2196/10961 (PMC6086930; doi:10.2196/10961)
Supplement: Multimedia Appendix 3 [file resprot_v7i7e10961_app3.pdf]

Multimedia Appendix 3: Summary of the Bibliometric Analysis of CLEF eHealth in 2013–2017

Organizations were not differentiated by their different departments in counting their number. Abbreviations: *academia* (ac), *conference paper* (CP), *government* (gov), *expression of interest* (EOI), *industry* (ind), *information retrieval* (IR), *journal paper* (JP), and *working note* (WN).

**Year**	**No. of EOIs (participating teams)**				**No. of Papers**			**No. of Authors**	**No. of Organizations**	**No. of Countries**	**No. of Citations**
	Task 1	***Task 2***	***Task 3***	***In total***	***WNs***	***CPs***	***JPs***		***In total (ac, gov, ind)***		
2012	-	-	-	-	16	0	0	50	35 (32, 2, 1)	8	20
2013	64 (22)	56 (5)	55 (9)	175 (34)	33	1	0	162	85 (74, 4, 7)	10	458
2014	50 (1)	79 (10)	91 (14)	220 (24)	28	1	0	107	69 (65, 0, 4)	22	273
2015	20 (2)	17 (7)	53 (12)	90 (20)	23	1	0	91	50 (47, 2, 1)	19	138
2016	25 (3)	33 (7)	58 (10)	116 (20)	23	1	0	113	73 (64, 6, 3)	16	110
2017	34 (11)	40 (14)	43 (7)	117 (32)	34	1	0	128	82 (75, 5, 2)	22	70
Other Papers	-	-	-	-	0	17	5	90	72 (70, 2, 0)	8	230
In total	193 (39)	225 (43)	300 (52)	718 (134)	157	22	5	741	466 (427, 21, 18)	33	1,299
