# Supplementary material for: Scholarly Influence of the Conference and Labs of the Evaluation Forum eHealth Initiative: Review and Bibliometric Study of the 2012 to 2017 Outcomes
Source: JMIR Res Protoc. 2018 Jul 27;7(7):e10961. doi: 10.2196/10961 (PMC6086930; doi:10.2196/10961)
Supplement: Multimedia Appendix 4 [file resprot_v7i7e10961_app4.pdf]

Multimedia Appendix 4: Summary of the Benchmark Results for the CLEF eHealth in 2013–2017

Notice that the tasks included some more subtasks – many of which resulted in statistically significant improvements in performance – but for the ease of reading this paper, our tabulation below is limited to the main tasks. Abbreviations: *accuracy* (A), *average precision* (AP), *error* (E = 1 - A), *precision* (P). "*" indicates that the measure value of the method was significantly better than the one for the next best method.

**Year**	**Task**	**Measure**		**Performance**				**Statistical Significance Testing**	
		Measure	***Value range***	***Best***	***2nd Best***	***3rd Best***	***Worst to illustrate the task difficulty***	***Test***	***Significant differences obtained between the methods***
2013	1a	F1	[0%, 100%]	75.0%*	73.7%*	70.7%*	42.8%	Random shuffling with *P* = .009	Yes
	1b	A	[0%, 100%]	58.9%*	58.7%*	54.6%*	0.6%	Random shuffling with *P* = .009	Yes
	2	A	[0%, 100%]	71.9%*	68.3%*	66.4%*	42.6%	Random shuffling with *P* = .009	Yes
	3	P@10	[0%, 100%]	51.8%	50.4%	48.4%	0.6%	Wilcoxon test with *P* = .04	Yes
2014	2a	A	[0%, 100%]	86.8%	85.4%	84.3%	76.9%	-	-
	2b	F1	[0%, 100%]	91.3%	67.1%	54.4%	19.0%	-	-
	3	P@10	[0%, 100%]	75.6%	75.5%	75.4%	6.0%	-	-

2015	1	E	[0%, 100%]	38.5%*	52.3%	52.8%*	95.4%	Wilcoxon test with *P* = .04	Yes
	2	F1	[0%, 100%]	75.6%	74.1%	70.4%	0.0%	-	-
	3	P@10	[0%, 100%]	53.9%	38.6%	38.0%	25.4%	-	-
2016	1	F1	[0%, 100%]	38.2%	37.4%*	34.5%*	0.0%	Wilcoxon test with *P* = .04	Yes
	2 (entity recognition)	F1	[0%, 100%]	74.9%*	70.2%*	69.9%*	12.6%	t-test with *P* < .001	Yes
	2 (cause of death)	F1	[0%, 100%]	84.8%*	84.4%*	75.2%*	55.4%	t-test with *P* < .001	Yes
	3	P@10	[0%, 100%]	-	-	-	-	.95 confidence intervals	-
2017	1	F1	[0%, 100%]	85.0%	85.0%	81.9%	0.11%	-	-
	2	AP	[0%, 100%]	31.8%	29.7%	29.3%	4.5%	-	-
	3	P@10	[0%, 100%]	-	-	-	-	.95 confidence intervals	-
